# Activity and Transcriptional Responses of Hepatopancreatic Biotransformation and Antioxidant Enzymes in the Oriental River Prawn *Macrobrachium nipponense* Exposed to Microcystin-LR

**DOI:** 10.3390/toxins7104006

**Published:** 2015-10-08

**Authors:** Julin Yuan, Xueqin Wang, Zhiming Gu, Yingying Zhang, Zaizhao Wang

**Affiliations:** 1 College of Animal Science and Technology, Northwest A & F University, Shaanxi Key Laboratory of Molecular Biology for Agriculture, Yangling 712100, Shaanxi, China; E-Mails: yuanjulin1982@hotmail.com (J.Y.); wangxueqin1988@hotmail.com (X.W.); yingyingz2008@hotmail.com (Y.Z.); 2 Zhejiang Institute of Freshwater Fisheries, Freshwater Fishery Healthy Breeding Laboratory of Ministry of Agriculture, Huzhou 313001, Zhejiang, China; E-Mail: guzhimin2006@hotmail.com

**Keywords:** microcystin-LR, biotransformation, antioxidant enzyme, gene expression

## Abstract

Microcystins (MCs) are a major group of cyanotoxins with side effects in many organisms; thus, compounds in this group are recognized as potent stressors and health hazards in aquatic ecosystems. In order to assess the toxicity of MCs and detoxification mechanism of freshwater shrimp *Macrobrachium nipponense*, the full-length cDNAs of the glutathione *S*-transferase (*gst*) and catalase (*cat*) genes were isolated from the hepatopancreas. The transcription level and activity changes in the biotransformation enzyme (glutathione *S*-transferase (GST)) and antioxidant enzymes (superoxide dismutase (SOD), catalase (CAT), glutathione peroxidase (GPx)) in the hepatopancreas of *M. nipponense* exposed to MC-LR (0.2, 1, 5, and 25 μg/L) for 12, 24, 72 and 96 h were analyzed. The results showed that the isolated full-length cDNAs of *cat* and *gst* genes from *M. nipponense* displayed a high similarity to other crustaceans, and their mRNAs were mainly expressed in the hepatopancreas. MC-LR caused significant increase of GST activity following 48–96 h (*p* < 0.05) and an increase in SOD activity especially in 24- and 48-h exposures. CAT activity was activated when exposed to MC-LR in 12-, 24- and 48-h exposures and then it was inhibited at 96-h exposure. There was no significant effect on GPx activity after the 12- and 24-h exposures, whereas it was significantly stimulated after the 72- and 96-h exposures (*p* < 0.05). The transcription was altered similarly to enzyme activity, but the transcriptional response was generally more immediate and had greater amplitude than enzymatic response, particularly for GST. All of the results suggested that MC-LR can induce antioxidative modulation variations in *M. nipponense* hepatopancreas in order to eliminate oxidative damage.

## 1. Introduction

Microcystins (MCs) are a family of cyclic peptide toxins produced by brackish and freshwater cyanobacteria of the genera *Anabaena*, *Anabaenopsis*, *Chroococcus*, *Microcystis*, *Nostoc*, and *Planktothrix* [1]. More than 80 structural variants have been identified, differing primarily in two *L*-amino acids. The major isoforms are microcystin-LR (MC-LR), microcystin-RR, and microcystin-YR [2]. Toxins are released into the surrounding medium during senescence and lysis of bloom cells, which affect many organisms, from terrestrial mammals to algae. In terrestrial mammals, MCs are selective for liver cells, irreversibly inhibiting serine/threonine protein phosphatases 1 (PP-1) and 2A (PP-2A), and causing disintegration of hepatocyte structure, apoptosis, necrosis, and internal hemorrhage in the liver which may lead to death by hemorrhagic shock [3,4,5]. Compared to terrestrial mammals, aquatic organisms are more frequently exposed to MCs. Studies with different fish species show that MCs can affect growth rate in *Rutilus rutilus* [6], histopathology in *Cyprinus carpio* [7], heart rate in *Salmo trutta* [8], blood index in *Hypophthalmichthys molitrix* and *Aristichthys nobilis* [9] and behaviour in *Odontesthes bonariensis* [10]. In aquatic invertebrate species, the effects of MCs mainly refer to the alteration of survival rate and feeding behavior of mussels [11] and bivalves [12], and the irreversible inhibition of serine/threonine protein phosphatases PP-1 and -2A is described in zooplankton [13]. In addition, several reports put forward that oxidative stress is also a toxicological consequence of the exposure to MCs in different aquatic organisms [14,15,16]. MCs could trigger excess reactive oxygen species (ROS), which result in lipid peroxidation (LPO), protein oxidation and DNA damage [16,17,18]. It is well known that aquatic organisms have developed a physiological antioxidant system, which involves antioxidant enzymes, such as superoxide dismutase (SOD), catalase (CAT), and glutathione peroxidase (GPx) and some non-enzymatic antioxidant molecule such as glutathione (GSH) [15,19]. These systems work together to eliminate the endogenous ROS (e.g., hydroxy radical, superoxide anion). Otherwise, GSTs, the most important enzymes for detoxifying MCs, are involved in the formation of a microcystin–glutathione conjugate, which enhances water solubility and has been detected in several aquatic organisms, including the crustacean *Daphnia magna* [20]. However, because of short-lived ROS in organs, the defense mechanism of aquatic invertebrate species induced by MCs still remains obscure and further studies are needed. The freshwater shrimp *Macrobrachium nipponense* is an important economic species bred widely in the Lake Tai region of China. The use of water contaminated with cyanobacteria in *M. nipponense* farms induced the obvious lower field production, which had caused enormous economic loss in recent years. In the present study, biotransformation and antioxidant enzymatic activity (GST, SOD, CAT and GPx) and transcriptional responses were monitored during 12-, 24-, 72- and 96-h exposure to assess the toxicity of MC-LR and the aquatic invertebrate species’ defense mechanism.

## 2. Materials and Methods

### 2.1. Animals and Chemicals

One-year-old adult *M. nipponense* (weight, 4.4 ± 0.2 g) were bought from Huzhou fish farm (Zhejiang, China) and raised in 50 L glass aquaria with aerated water, a controlled temperature of 25 ± 2 °C, and the photoperiod was a 14 h:10 h light/dark cycle. Males were chosen to minimize possible interfering effects of sex. MC-LR (purity ≥ 98%) was purchased from Alexis Biochemicals (Lauseanne, Switzerland).

### 2.2. MC-LR Exposure

*M. nipponense* were exposed to MC-LR or a solvent control (0.05% methanol, *v/v*) in a 20 L glass tank for 12, 24, 48, 72 and 96 h (15 individuals per tank in triplicate and approximately 3.3 g shrimp/L). The MC-LR doses were 0.2, 1, 5, and 25 μg/L, according to environmental content of MCs. Half of the exposure solution was changed every day in order to guarantee the concentration of MC-LR and water quality. They were fed with commercial pellet food once a day. Water quality was regularly monitored and no differences were recorded between tanks during the entire experimental period. No crayfish died during the exposure period. *M. nipponense* in each group were immediately sacrificed following exposure, and tissues were dissected, frozen in liquid nitrogen, and maintained at −80 °C until use.

### 2.3. Sampling, RNA Isolation, and Reverse Transcription (RT)

*M. nipponense* hepatopancreas, muscle, stomach, intestine, and gill tissues were assayed for *cat* and *gst* mRNAs. Tissue samples for cDNA cloning and quantitative real-time polymerase chain reaction (qRT-PCR) analysis were homogenized in TRIZOL reagent (Invitrogen, Carlsbad, CA, USA), total RNA was extracted as described by Wang *et al.* [21], and treated with RNase-free DNase I (Fermentas, Burlington, ON, Canada) to remove genomic DNA contamination. Total RNA concentrations were calculated at an absorbance of 260 nm. Total RNA quality was verified on 1% agarose gels by visual inspection of the 18 S and 28 S ribosomal RNA bands and by the A260 nm/A280 nm ratio (range 1.90–2.05) measured with a Nanodrop spectrophotometer (Thermo Electron Corp., Waltham, MA, USA). The cDNAs were synthesized from 5 μg total RNA with M-MLV reverse transcriptase (Invitrogen) and the oligo (dT)_18_ primer in a 20 μL final volume.

### 2.4. Cloning of Full-Length Cat and Gst cDNAs from *M. nipponense*

Partial cDNA fragments for the two target genes in *M. nipponense* were amplified using primers designed from the conserved regions of their counterparts in other crustaceans (Table S1). PCR was performed with 2 μL of the cDNA template synthesized by RT-PCR in 20 μL containing 5 pmol of each specific primer and 1 unit of Taq DNA polymerase (TaKaRa Bio, Shiga, Japan). The purified PCR products of these cDNA fragments were inserted into the pMD_18_-T plasmid using a TA cloning kit (TaKaRa Bio) following the manufacturer’s instructions. Three confirmed recombinant plasmid clones for each gene were sequenced separately by Genscript Corp. (Nanjing, China), using the ABI 3730 automated DNA sequencer (BigDye Terminator Chemistry) and the BigDye Terminator Cycle Sequencing Kit (PE Biosystems, Foster City, CA, USA). The nucleotide sequences were determined for both sense and antisense strands of the plasmid inserts. The 3′ and 5′ ends of both cDNAs were obtained with several pairs of gene-specific primers designed for the target genes to overlap the sense or antisense regions of their amplified partial fragments sequenced above. The 5′ and 3′ rapid amplification of cDNA ends (RACE) procedures were performed with the RACE cDNA Amplification Kit (Invitrogen), using total RNA and following the manufacturer’s instructions, respectively. The nested 5′- and 3′-RACE PCR products of the expected size were processed and sequenced as described above. The *cat and gst* full-length cDNAs were assembled by aligning the partial cDNA fragments and the 5′- and 3′-RACE fragments using SeqMan in Lasergene software (DNASTAR, Madison, WI, USA).

### 2.5. Protein Alignment and Phylogenetic Analyses

The putative amino acid sequences of the *M. nipponense* *cat* and *gst* genes were aligned with their counterparts from other species using the Megalign program in Lasergene software. We aligned diverse invertebrate *cat* and *gst* genes at the amino acid level using the ClustalX (1.83) sequence alignment program to establish phylogenetic trees for the two *M. nipponense* genes [22]. The neighbor-joining algorithm method [23] in the Mega 4.0 program was used to construct the phylogenetic trees [24]. Bootstrap analyses were conducted using 1000 replicates.

### 2.6. qRT-PCR

The qRT-PCR analysis was performed on a CFX96 real-time PCR detection system (Bio-Rad, Hercules, CA, USA) thermocycler with the SYBR Premix ExTaq II kit (TaKaRa Bio). The qRT-PCR reactions were carried out in a final volume of 25 μL using 1 × SYBR Premix Ex Taq, 0.4 μM of each primer, and 2.5 μL RT reaction solution. The reactions were denatured at 95 °C for 30 s, followed by 40 cycles of denaturation at 95 °C for 5 s, and annealing at 60 °C for 30 s. To assess the specificity of each amplicon, a melting curve analysis was performed at the end of each PCR thermal profile. CFX Manager software (Bio-Rad) was used to analyze SYBR Green I density and to determine the threshold cycle (Ct) value. All samples were run in triplicate. qRT-PCR efficiency (E) was calculated from the given slopes using CFX Manager software and a 10-fold diluted cDNA sample series with five dilution points measured in triplicate. E was determined using the equation: E = 10$\frac{-1}{\text{slope}}$ [25].

### 2.7. Tissue Distribution of the Cat and Gst Genes

qRT-PCR was conducted with male *M. nipponense* hepatopancreas, intestine, gill, stomach, and muscle tissues to analyze tissue distribution of the *cat* and *gst* transcripts, with the β*-actin* gene as the endogenous control [26]. The qRT-PCR primers are listed in Table S1. The primers were tested during normal PCR amplification, and the PCR products were visualized on a 1.5% agarose gel before qRT-PCR to verify primer specificity. The relative changes in mRNA levels of the two genes in different tissues were calculated using the 2^−ΔΔCt^ method [27]. The analysis was performed on tissues from nine males. All data are expressed as mean ± standard deviation (SD).

### 2.8. Enzyme Activity Assays Following MC-LR Exposure

Nine hepatopancreatic samples from each group were defrosted twice and homogenized on ice with 10 volumes of cold buffer (250 mM sucrose, 5 mM Tris-HCL, and 0.1 mM edetic acid-2Na; pH 7.5). The homogenate was centrifuged at 13,000 g and 4 °C for 10 min to obtain the supernatant for the assays. Protein concentration was determined using a commercial protein assay kit (Nanjing Jiancheng Bioengineering Institute). GST, SOD, CAT, and GPx activities were measured using commercial kits (Nanjing Jiancheng Bioengineering Institute, Nanjing, China). One unit (U) of GST, CAT, and GPx activity was defined as the amount of enzyme consuming 1 μmol of substrate or generating 1 μmol of product/min/mg soluble protein (U/mg prot). One U of SOD activity was defined as the amount of enzyme required to inhibit the oxidation reaction by 50% and was also expressed as U/mg prot.

### 2.9. mRNA Expression of the Enzymes Following MC-LR Exposure

The gene expression patterns of hepatopancreatic *gst*, Cu/Zn-*sod*, *cat*, and *gpx* were detected by qRT-PCR in *M. nipponense* after exposure to 0.2–25 μg/L MC-LR for 96 h. Each transcript was analyzed in 9 individuals. The β*-actin* gene was used as an endogenous control. Relative mRNA levels were calculated using the 2^−ΔΔCt^ method and the formula F = 2^−ΔΔCt^, ΔΔCt = (C_t, target gene_ − C_t, β*-actin*_) _MC-LR_ − (C_t, target gene_ − C_t, β*-actin*_) _control_ [27]. Data are expressed as mean ± SD. Statistical differences were tested with analysis of variance and the least significant difference test.

## 3. Results

### 3.1. Cloning and Molecular Characterization of the Cat and Gst Gene cDNAs

The full-length cDNA sequences of *cat* (GenBank accession No. KC485002) and *gst* (GenBank accession No. KC485003) genes were determined by the RT-PCR and RACE methods. The *cat* cDNA was 1699 bp and contained a 1554 bp open reading frame (ORF) encoding a 518 amino acid polypeptide with a theoretical pI of 6.66 and a calculated molecular mass of 58.57 kDa and 135 bp 5′-untranslated region (UTR) (Figure S1). Meanwhile, the *gst* cDNA was 1122 bp and contained a 651 bp ORF encoding a 217 amino acid polypeptide with a theoretical pI of 5.35 and a calculated molecular weight of 25.45 kDa, 173 bp 5′-UTR and 298 bp 3′-UTRs with a polyadenylation signal (AATAAA) (Figure S2).

### 3.2. Homology and Phylogenetic Analyses of the Putative Cat and Gst Amino Acid Sequences

The putative CAT of *M. nipponense* contains NADPH binding site, proximal heme-ligand signature, and proximal active site signature (Figure S3). The amino acid sequence identities of *M. nipponense* CAT with its counterparts from other crustaceans were more than 80%. The *M. nipponense* GST protein sequence showed 53.3%–72.1% similarity with the GST mu class from different organisms and had several conserved GSH binding sites and substrate binding sites (Figure S4).

**Figure 1:**
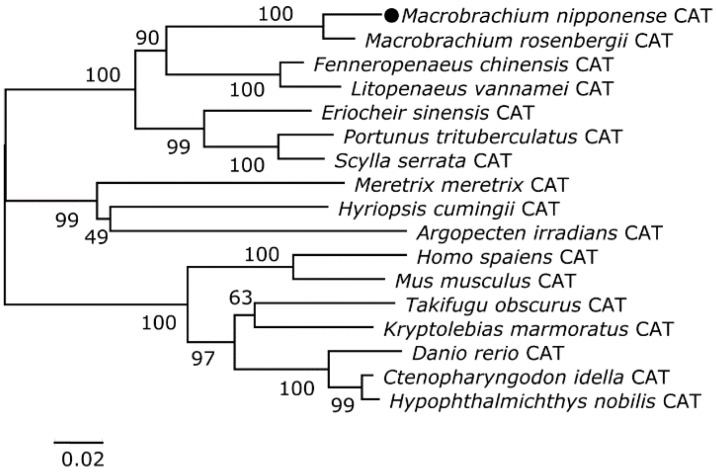
The phylogenetic tree was constructed based on a deduced amino acid alignment for *M. nipponense* CAT and its counterparts in other species using the neighbor-joining method. The accession numbers for the sequences are as follows: *Macrobrachium rosenbergii*, HQ668089; *Fenneropenaeus chinensis*, EU102287; *Litopenaeus vannamei*, JX162772; *Eriocheir sinensis*, GU361618; *Portunus trituberculatus*, FJ152102; *Scylla serrata*, GQ892832; *Meretrix meretrix*, JQ005875; *Hyriopsis cumingii*, HM188565; *Argopecten irradians*, HQ025801; *Homo sapiens*, NM_001752; *Mus musculus*, NM_009804; *Takifugu obscurus*, EF667052; *Kryptolebias marmoratus*, EU116026; *Danio rerio*, NM130912; *Ctenopharyngodon idella*, FJ560431; and *Hypophthalmichthys nobilis*, HM564034. Bootstrap values (%) are indicated (1000 replicates). Scale bar indicates 0.02 expected amino acid substitutions per site.

**Figure 2:**
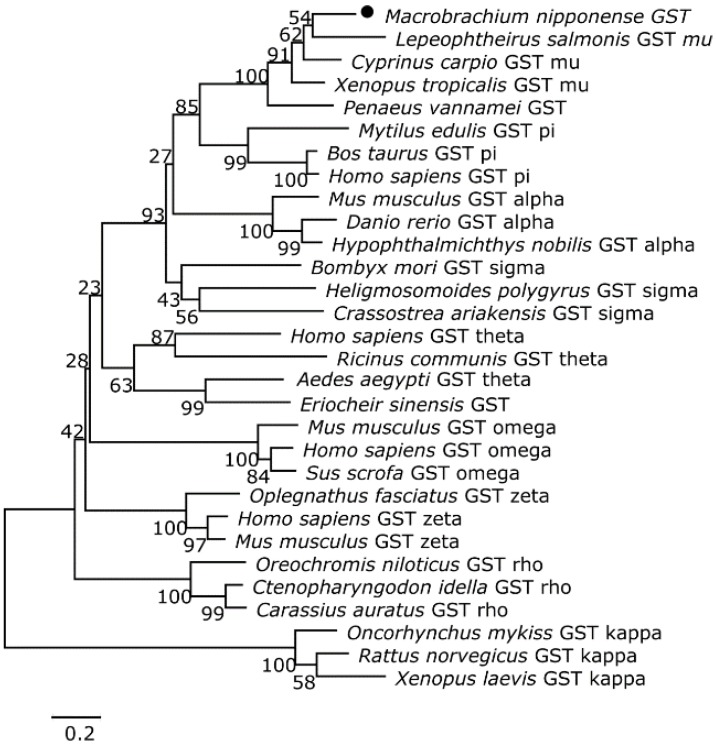
The phylogenetic tree was constructed based on a deduced amino acid alignment for *M. nipponense* GST and its counterparts in other species using the neighbor-joining method. The accession numbers for the sequences are as follows: *Lepeophtheirus salmonis* GST mu, BT121603; *Cyprinus carpio* GST mu, DQ411312; *Xenopus tropicalis* GST mu, NM001004964; *Penaeus vannamei* GST, AY573381; *Mytilus edulis* GST pi, AY557404; *Bos taurus* GST pi, NM_177516; *Homo sapiens* GST pi, BC010915; *Mus musculus* GST alpha, BC012639; *Danio rerio* GST alpha, NM_213394; *Hypophthalmichthys nobilis* GST alpha, EF100902; *Bombyx mori* GST sigma, NM_001043529; *Heligmosomoides polygyrus* GST sigma, AF128959; *Crassostrea ariakensis* GST sigma, EU908270; *Homo sapiens* GST theta, NM_000853; *Ricinus communis* GST theta, XM002535811; *Aedes aegypti* GST theta, AY819712; *Eriocheir sinensis* GST, GQ325712; *Mus musculus* GST omega, NM_010362; *Homo sapiens* GST omega, NM_004832; *Sus scrofa* GST omega, NM_004832; *Oplegnathus fasciatus* GST zeta, GU938679; *Homo sapiens* GST zeta, NM_145870; *Mus musculus* GST zeta, NM_010363; *Oreochromis niloticus* GST rho, EU107284; *Ctenopharyngodon idella* GST rho, EU107283; *Carassius auratus* GST rho, EU527005; *Oncorhynchus mykiss* GST rho, NM_001165133; *Rattus norvegicus* GST kappa, NM_181371; and *Xenopus laevis* GST kappa, NM_001086734. Bootstrap values (%) are indicated (1000 replicates). Scale bar indicates 0.2 expected amino acid substitutions per site.

Two phylogenetic trees were constructed from the amino acid sequence alignments for CAT and GST in crustaceans, fish, and mammals using the neighbor-joining method to better understand the positions of *M. nipponense* CAT and GST in the evolutionary history of the respective proteins. *M. nipponense* CAT was more similar to its counterpart in *Macrobrachium rosenbergii* (Figure 1). GST was more similar to its counterpart in *Lepeophtheirus salmonis* than those of other crustaceans and fish (Figure 2).

### 3.3. Tissue Distributions for Cat and Gst mRNAs

**Figure 3:**
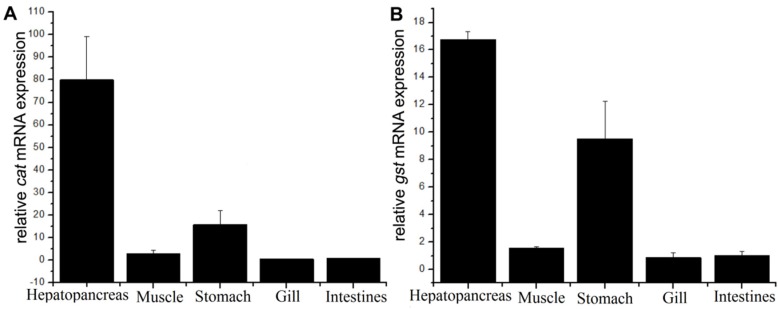
mRNA tissue distributions of *cat* (**A**) and *gst* (**B**) in *M. nipponense* tissues of hepatopancreas, muscle, stomach, gill and intestines analyzed by quantitative reverse transcription-polymerase chain reaction analysis. The values were calibrated with the β*-actin* endogenous control. Values are expressed as mean ± standard deviation (*n* = 9).

The *cat* and *gst* tissue distributions in *M. nipponense* are shown in Figure 3. Both *cat* and *gst* mRNAs were expressed predominantly in the hepatopancreas. Meanwhile, *cat and gst* mRNAs were expressed at low level in other tissues with decreasing order of stomach, muscle, intestines and gill. The *cat* and *gst* mRNA levels in hepatopancreas were 80.1-fold and 16.8-fold higher than in intestine, respectively.

### 3.4. Effect of MC-LR on Biotransformation Enzyme and Antioxidant Enzymes

**Figure 4:**
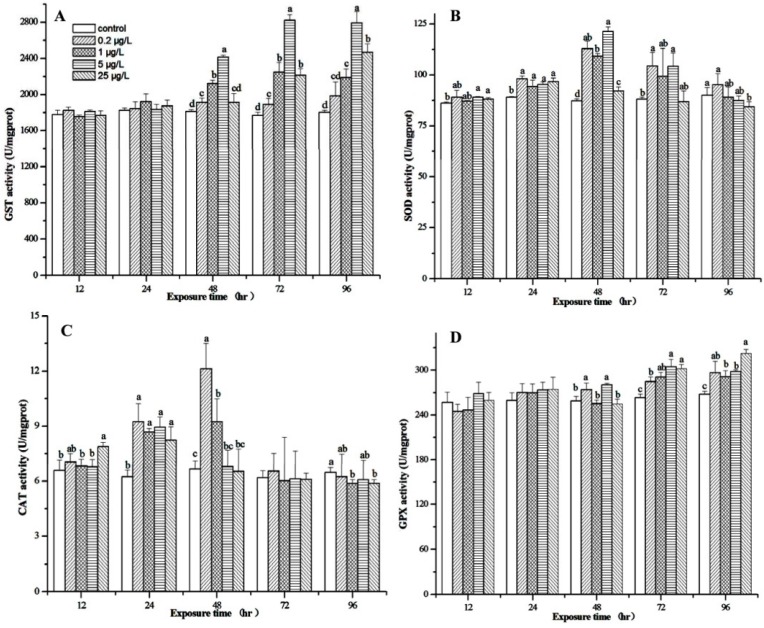
Glutathione *S*-transferase (GST) (**A**); superoxide dismutase (SOD) (**B**); catalase (CAT) (**C**); and glutathione peroxide (GPx) (**D**) enzyme activities in the hepatopancreas of male *M. nipponense* after MC-LR exposure for 12, 24, 48, 72 and 96 h at different concentrations. Values are expressed as mean ± standard deviation. Different lowercase letters indicate significant differences (*p* < 0.05), and different capital letters indicate highly significant differences (*p* < 0.01).

MC-LR had no effect on GST activity after the 12- and 24-h exposures (Figure 4A), but GST activity increased significantly following MC-LR exposure at four concentrations for 48–96 h, except MC-LR exposure at 1 μg/L for 48 h and 0.2 μg/L for 96 h (Figure 4A).

MC-LR at 5 and 25 μg/L caused a significant increase in SOD activity after the 12-h exposure, compared with control (*p* < 0.05, Figure 4B). At the same time, 0.2–25 μg/L MC-LR all significantly stimulated SOD activity in the 24- and 48-h exposures (1.06–1.38 times, *p* < 0.05, Figure 4B). Amounts of 0.2 and 5 μg/L MC-LR also significantly increased SOD activity in the 72-h exposure (*p* < 0.05, Figure 4B), but 1 and 25 μg/L MC-LR did not increase SOD activity. However, in the 96-h exposure, 25 μg/L MC-LR significantly inhibited SOD activity, while MC-LR at 0.2–5 μg/L had no significant effect (*p* < 0.05, Figure 4B).

In 12-h exposure, only 25 μg/L MC-LR significantly stimulated CAT activity, while 0.2–5 μg/L MC-LR had no significant effect (Figure 4C). In 24-h exposure, MC-LR at 0.2–25 μg/L all significantly increased CAT activity (Figure 4C). In 48-h exposure, 0.2 and 1 μg/L MC-LR caused 1.8- and 1.4-fold significant increases in CAT activity, respectively (*p* < 0.05, Figure 4C), while 5 and 25 μg/L MC-LR had no significant effect. For 72-h exposure, all MC-LR treatments did not affect CAT activity. MC-LR at 1 and 25 μg/L significantly suppressed CAT activity after the 96-h exposure, whereas 0.2 and 5 μg/L MC-LR had no significant effect (Figure 4C).

It was interesting that none of the MC-LR concentrations had a significant effect on GPx activity in the 12- and 24-h exposures (Figure 4D). MC-LR at 0.2 and 5 μg/L significantly enhanced GPx activity after the 48-h exposure, whereas 1 and 25 μg/L MC-LR did not change GPx activity. In the 72- and 96-h exposures, MC-LR at 0.2–25 μg/L all significantly stimulated GPx activity (*p* < 0.05, Figure 4D).

### 3.5. Expression Profiles of M. nipponense Gst, Cu/Zn-sod, Cat, and Gpx in the Hepatopancreas After MC-LR Exposure

**Figure 5:**
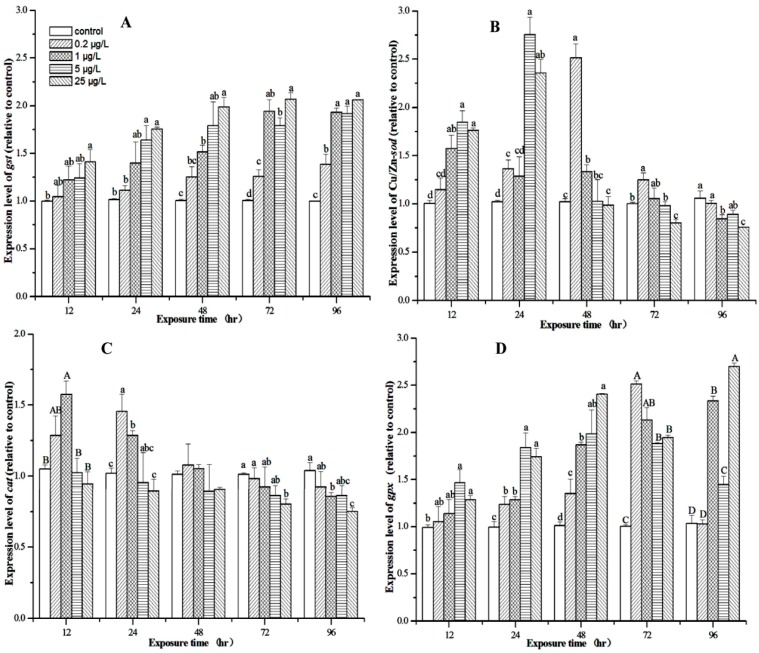
Relative glutathione *S*-transferase (*gst*) (**A**); Cu/Zn-superoxide dismutase (*sod*) (**B**); catalase (*cat*) (**C**); and glutathione peroxidase (*gpx*) mRNA expression (**D**) in the hepatopancreas of *M. nipponense* measured by quantitative reverse transcription-polymerase chain reaction analysis after acute microcystin (MC)-LR exposure for 12, 24, 48, 72 and 96 h. The values were calibrated with the endogenous control β*-actin*. Each transcript abundance value is expressed as mean ± standard deviation (*n* = 9 for each value). Different lowercase letters indicate significant differences (*p* < 0.05), and different capital letters indicate highly significant differences (*p* < 0.01).

The changes in *gst*, Cu/Zn-*sod*, *cat* and *gpx* mRNA expression are shown in Figure 5. The *gst* mRNA expression was significantly up-regulated 1.41-fold by 25 μg/L MC-LR after the 12-h exposure (*p* < 0.05) (Figure 5A), whereas MC-LR at the lower concentrations (0.2–5 μg/L) had no significant effect. In 24-h exposure, MC-LR at 5 and 25 μg/L significantly elevated *gst* transcription 1.62 and 1.73-fold, respectively (Figure 5A), while 0.2 and 1 μg/L MC-LR did not significantly affect it. In 48-h exposure, MC-LR at 1–25 μg/L caused 1.51–1.98-fold significant increases in *gst* transcription. In 72- and 96-h exposures, MC-LR at 0.2–25 μg/L all significantly up-regulated *gst* expression (1.25–2.06-fold, 1.39–2.07-fold, respectively, *p* < 0.05, Figure 5A).

In 12-h exposure, MC-LR at 1–25 μg/L caused 1.57–1.84-fold significant increase in Cu/Zn-*sod* transcript (*p* < 0.05), while 0.2 μg/L MC-LR had no significant effect (Figure 5B). After 24-h exposure, MC-LR at 0.2, 5, and 25 μg/L significantly elevated Cu/Zn-*sod* transcription (1.33–2.69-fold, *p* < 0.05), whereas 1 μg/L MC-LR had no significant effect. In 48-h exposure, Cu/Zn-*sod* expression was significantly up-regulated for 2.46 and 1.3 fold by 0.2 and 1 μg/L MC-LR, respectively, whereas MC-LR at higher concentrations (5 and 25 μg/L) had no significant effect on Cu/Zn-*sod* transcription. In 72-h exposure, Cu/Zn-*sod* was significantly up-regulated and down-regulated by 0.2 and 25 μg/L MC-LR, respectively. However, 1 and 5 μg/L MC-LR had no effect. Both 1 and 25 μg/L MC-LR significantly down-regulated Cu/Zn-*sod* expression after the 96-h exposure and MC-LR at 0.2 and 5 μg/L tended to inhibit Cu/Zn-*sod* transcription (Figure 5B).

After 12-h exposure, *cat* expression was significantly up-regulated by the 1 μg/L MC-LR for 1.57-fold (*p* < 0.01, Figure 5C), while MC-LR at 0.2, 5 and 25 μg/L all had no significant effect. In 24-h exposure, MC-LR at 0.2 and 1 μg/L significantly stimulated *cat* mRNA expression (1.4 and 1.2-fold, *p* < 0.05, respectively), whereas MC-LR at higher concentrations (5 and 25 μg/L) tended to inhibit its expression. In 72-h treatment, *cat* expression was significantly down-regulated by 25 μg/L MC-LR (0.8-fold, *p* < 0.05), and there was inhibitory tendency for *cat* expression in MC-LR at lower concentrations (0.2–5 μg/L). After 96-h exposure, MC-LR at 1 and 25 μg/L resulted in 0.82- and 0.72-fold decreases of *cat* transcription, respectively (*p* < 0.05, Figure 5C), while MC-LR at 1 and 5 μg/L tended to inhibit its expression. However, 48-h MC-LR at 4 concentrations did not affect *cat* expression.

After 12-h exposure, MC-LR at 5 and 25 μg/L significantly increased *gpx* mRNA expression for 1.48- and 1.29-fold, respectively (*p* < 0.05, Figure 5D), while MC-LR at lower concentrations had no effect. During 24-h exposure, MC-LR at 0.2–25 μg/L all significantly up-regulated *gpx* mRNA expression (1.24–1.85-fold, *p* < 0.05). After 48-h exposure, 1–25 μg/L MC-LR significantly stimulated *gpx* expression 1.85–2.38-fold, and 0.2 μg/L MC-LR tended to increase *gpx* expression. During 72-h exposure, MC-LR at 0.2–25 μg/L caused 1.87–2.5-fold, significant increases in *gpx* transcript (*p* < 0.01). MC-LR at 1–25 μg/L significantly increased *gpx* transcript 1.4–2.6 fold after 96-h exposure (*p* < 0.01) (Figure 5D), while 0.2 μg/L MC-LR did not affect its expression.

## 4. Discussion

In this study, CAT and GST cDNA sequences cloned from *M. nipponense* were presented, and sequence alignment analysis demonstrated this CAT shared high identity (>80%) with catalase from other crustaceans. The predicted size of the crayfish CAT polypeptide was 58.57 kDa, very close to swimming crab *Portunus trituberculatus* (58.57 kDa) [28], Chinese shrimp *Fenneropenaeus chinensis* (58.8 kDa) [29] and white shrimp *Litopenaeus vannamei* (57.6 kDa) [30]. The *M. nipponense* catalase proximal heme-ligand signature sequence (RLFSYNDTH) and the proximal active site signature (FDRERIPERVVHAKGAGA) were highly conserved with other species [31,32], which suggested that this CAT should have the same function with other species due to the conserved sequences and motif. Two ligand binding sites were present in GST. The G-sites were highly specific for glutathione binding sites and contained 11 highly conserved amino acids: Tyr^7^, Arg^13^, Trp^38^, Lys^46^, Gln^53^, Leu^54^ Pro^55^, Gln^66^, Ser^67^, Glu^98^ and Asp^99^ (alignment positions in GST of *M. nipponense* 7, 11, 46, 50, 59, 60, 61, 72, 73, 101 and 102, respectively) [33,34]. The second ligand binding site, H-sites, interacting with electrophilic xenobiotic substrates, contained eight conserved residues: Tyr^7^, Phe^8^, Val^10^, Arg^13^, Val^104^, Tyr^108^, Asn^204^ and Gly^205^ [35]. Most of these characteristic amino acids were found in GST of *M. nipponense*, with the exception of Asn^53^, Asn^99^ in the G-sites and Try^8^, Ile^10^ in the H-sites, which were respectively substituted for Gln^53^, Asp^99^ and Phe^8^, Val^10^. The predominant *cat* and *gst* expression in the hepatopancreas agreed with previous studies on white shrimp, *Litopenaeus vannamei* [36] and tilapias [37]. Therefore, the hepatopancreas was selected to detect *cat* and *gst* gene expression following exposure to MC-LR as the main antioxidative tissue of *M. nipponense*.

The rate or amounts of reactive oxygen species (ROS) generation can be induced by the presence of a wide range of natural and anthropogenic xenobiotics or toxins [38]. Much evidence suggested that MCs-induced oxidative damage could be a result of ROS generation [39,40]. The SOD-CAT system provides the first defense against oxygen toxicity. SOD catalyzes the dismutation of the superoxide radical to molecular oxygen and hydrogen peroxide [41]. Increased SOD activity is part of the adaptive mechanism to oxidative stress [42]. In the present study, the SOD activity was all stimulated by MC-LR at 0.2–25 μg/L in the early stage of exposure (12–72 h), and with an exposure duration from 12–48 h, the increase of SOD activity was shown to be time-dependent at almost four concentrations. The results indicated that the antioxidant defense system of *M. nipponense* was rapidly activated after being exposed to MC-LR and had high efficiency to restore oxidative balance. In 96-h exposure, MC-LR tended to inhibit SOD activity, especially at 25 μg/L, which was in line with the results in a previous study on freshwater mussel *Dreissena polymorpha* [43]. It is suggested that the exposure duration or concentration for MC-LR is not sufficient to provoke an increase in activity of the species. Pigeolet *et al.* [44] demonstrated that SOD was itself susceptible to oxidation and was suppressed by peroxides and oxygen derived free radicals. Therefore, in the present study, the high level of ROS at the end of exposure (96-h) might be detrimental to SOD activity. CAT is a key enzyme to remove the H_2_O_2_ produced by SOD catalyzing dismutation of highly reactive superoxide anions [45]. In the present study, the CAT activity was increased by MC-LR in the early stage of exposure (12–48 h), which indicated an activation of antioxidant defense system and non-lagged direct or indirect ROS production upon MC-LR exposure. However, at the end of exposure (96-h), the CAT activity had decreased with significant suppression at 1 and 25 μg/L. In estuarine crab *Chasmagnathus granulates*, seven-day MC exposure also caused a decrease of CAT activity [46]. Previous study suggested that peroxides and oxygen derived free radicals inhibited CAT activity [44,47]. So, we speculate that the CAT might be suppressed by extra ROS at the end of exposure.

GSTs are a group of multifunctional dimeric proteins involved in cellular detoxification reaction, catalyzing the enzymatic conjugation of GSH to toxic compounds, including MCs [48]. The reaction will neutralize the electrophilic sites of MCs, increasing their water solubility and favoring their excretion [49]. In mussel *Dreissena polymorpha*, crustacean *Daphnia magna* and zebrafish *Danio rerio*, MC-LR glutathione conjugates were formed enzymatically via soluble GST [50], which suggested that GSTs played a key role in detoxification of MC-LR. GSTs contain two forms: the soluble form (sGST) and the membrane form (mGST). The sGST is found in the cell cytosol and mGST is bound to membrane of microsome, peroxisome, mitochondria and endoplasmic reticulum. In the present study, the sGST and mGST were not separated, so the GST activity signifies a combination of sGST and mGST activities. In the freshwater shrimp *Palaemonetes argentines* exposed to MCs, activity of sGST and mGST had a similar regulatory trend at any exposure time point [51]. So, we speculate that modulation of sGST in hepatopancreas of *M. nipponense* upon MC-LR may have a similar trend to the regulation of total GST activity in the present study. In the present study, mu-GST activity was significantly stimulated by MC-LR at 0.2–25 μg/L from 48–96 h, which was consistent with the results of studies on other aquatic organisms such as crab and fish [52,53].

The function of GPx is to reduce free hydrogen peroxide to water and to reduce lipid hydroperoxides to their corresponding alcohols. GPx activity in the *M. nipponense* hepatopancreas was not significantly affected during the early stage of MC-LR exposure (12–24 h), while it was significantly stimulated by MC-LR even at 0.2 μg/L in the later stage of exposure (72–96 h). It seems that the enzymatic activity stimulation for GPx and CAT in response to MC-LR complements each other in the early and later stage of exposure. It suggested that the activation of GPx by MC-LR exposure might lag behind CAT.

Although increasing numbers of researches have focused on the oxidative stress responses arising from MCs, most of them were limited and mainly examined the changes in antioxidative enzymatic activity. Recently, differential expression of the genes encoding these antioxidant proteins has been used to detect biological toxicity [54,55]. In the present study, the transcriptional changes for *gst*, Cu/Zn-*sod*, *cat*, and *gpx* genes generally followed a similar trend to the changes in the respective enzyme activities in *M. nipponense* exposed to MC-LR. *gst*, Cu/Zn-*sod*, *cat*, and *gpx* transcription generally changed with higher amplitude compared with the activities of their respective enzymes during MC-LR exposure, suggesting that changes in transcription of antioxidant enzymes could serve as sensitive molecular biomarkers for exposure to MC-LR in *M. nipponense*, particularly for *gst* genes. In several exposure scenarios, the enzyme transcriptional changes were inconsistent with the changes in enzyme activities, such as CAT after the 12- and 24-h MC-LR exposures at 25 μg/L and GST and GPx after the 12- and 24-h MC-LR exposures at 5 and 25 μg/L. This discrepancy suggested that the mRNA level of an enzyme signified a snapshot of its activity at any given time point, whereas enzyme activity may be regulated post-transcriptionally [56]. A study on common carp, *Cyprinus carpio*, exposed to MC showed that a time window probably exists between transcription and translation, as enzyme activity and mRNA expression were detected simultaneously [57].

## 5. Conclusions

In the present study, the full lengths of *gst* and *cat* cDNAs were firstly isolated in *M. nipponense*, and their putative amino acid sequences displayed high similarity to their respective counterparts of crustacean species. The mRNAs of *gst* and *cat* were mainly expressed in the hepatopancreas. The activity and transcriptional level variations of biotransformation enzyme (GST) and antioxidant enzymes (SOD, CAT, and GPx) indicated that MC-LR induced ROS production and crayfish made corresponding reaction to restore oxidative balance. The transcriptional response was generally more immediate and with greater amplitude than the enzyme activity responses, particularly for GST, which suggested that transcription of these genes could be a sensitive molecular biomarker for acute MC-LR exposure.

## Supplementary Materials

Supplementary materials can be accessed at: http://www.mdpi.com/2072-6651/7/10/4006/s1.
