# Supplementary material for: Activity and Transcriptional Responses of Hepatopancreatic Biotransformation and Antioxidant Enzymes in the Oriental River Prawn Macrobrachium nipponense Exposed to Microcystin-LR
Source: Toxins (Basel). 2015 Oct 8;7(10):4006–22. doi: 10.3390/toxins7104006 (PMC4626717; doi:10.3390/toxins7104006)
Supplement: Supplementary file 1 [file toxins-07-04006-s001.pdf]

Supplementary Information

Table S1. Primers used for fragment cloning, rapid amplification of cDNA ends, and quantitative real-time polymerase chain reaction analyses of *M. nipponense* catalase (*cat*), glutathione *S*-transferase (*gst*), Cu/Zn-superoxide dismutase (*sod*), and glutathione peroxidase (*gpx*).

Primer Name	Primer Sequence (5′ to 3′)	Remark	Reference
cat-F<br>*cat*-R	CGCAGGTGCCTTTGGATAC<br>GCCTTCTCCGCACTCAAAT	*cat* fragment cloning	
cat-5gsp1<br>*cat*-5gsp2	GTCTCGGATAAAGAACACGGGT<br>AGCAATAGGTGTCTTCTTCCCAA	*cat* 5′-RACE	
cat-3gsp1<br>*cat*-3gsp2	CGTGTTCTTTATCCGAGACCCT<br>ACCAGGTCACCATCCTTTTCTC	*cat* 3′-RACE	
gst-F<br>*gst*-R	CTTCCCCAACCTTCCCTACTA<br>CGACGCCATGTACTTCTTGAT	*gst* fragment cloning	
gst-5gsp1<br>*gst*-5gsp2	CACCAGCATACCAGGGTTGTT<br>TTCTCAGATTCAGTCTTGCCACA	*gst* 5′-RACE	
gst-3gsp1<br>*gst*-3gsp2	TGAATCTGAGAAAGTCCGTGTTG<br>AGCAATAGGTGTCTTCTTCCCAA	*gst* 3′-RACE	
5′-RACE Outer Prime<br>5′-RACE Inner Primer	CATGGCTACATGCTGACAGCCTA<br>CGCGGATCCACAGCCTACTGATGATCAGTCGATG	*cat* gene walking	
3′-RACE Outer Primer<br>3′-RACE Inner Primer	TACCGTCGTTCCACTAGTGATTT<br>CGCGGATCCTCCACTAGTGATTTCACTATAGG	*gst* gene walking	
cat-qRT-F<br>*cat*-qRT-R	GAACTGGGATTTGGTTGGCA<br>GGTCCGAGAAAAGGATGGTG	*cat* qRT-PCR	This study
gst-qRTF<br>*gst*-qRTR	GAACTGGGATTTGGTTGGCA<br>GGTCCGAGAAAAGGATGGTG	*gst* qRT-PCR	This study
Cu/Zn-*sod*-qRT-F<br>Cu/Zn-*sod*-qRT-R	AGTTTCAGCCGTCTGTTCG<br>CACAGTGCTTACATCACCCTTA	Cu/Zn-*sod* qRT-PCR	Accession number: JX045662
gpx-qRT-F<br>*gpx*-qRT-R	CCTGGCTTTCCCCTGTAACC<br>ACCGAGTCATCCGAAGGCA	*gpx* qRT-PCR	Accession number: HQ651155
β-actin-qRTF<br>*β-actin*-qRTR	AATGTGTGACGACGAAGTAG<br>GCCTCATCACCGACATAA	β-*actin* qRT-PCR	Sun *et al.* 2015 [26]

```
1 gaaaagtctgggagtgagtgaggtacgtgacacagacaacacagcagtcagtcacacagacggtacgttagtgtccggagtgaaggtaca
M P R D R A A E Q L N E F K K
91 ctttttaaaagtctcaagtgatctatccacggtcctatatccacgATGCCTCGCGACAGAGCTGCCGAGCAACTGAACGAGTTCAAGAAG
K Q T T P E V L T T S Q G C P L A D K L N S P T V G P R G P
181 AAGCAGACGACTCCTGAAGTCTTAACAACCTCGCAAGGATGTCCCCTGGCAGACAAGCTCAATTCCCCGACAGTGGGTCCCAGAGGTCCT
I L L Q D I Q L L D E M A H F D R E R I P E R V V H A K G A
271 ATCCTCCTGCAGGATATCCAACTCCTGGACGAGATGGCCCACTTCGACAGGGAGCGCATCCCGGAGAGAGTAGTGCACGCTAAGGGCGCT
G A F G H F E V T H D I T K Y C R A N I F S E I G K K T P I
361 GGTGCCTTTGGACACTTCGAAGTCACCCATGACATTACGAAGTATTGCAGAGCCAACATATTCAGTGAGATTGGGAAGAAGACACCTATT
A V R F S T V G G E S G S A D T A R D P R G F A V K F Y T E
451 GCTGTAAGATTCTCTACTGTAGGAGGTGAAAGTGGTTCTGCAGACACGGCTAGGGATCCTCGTGGCTTCGCTGTCAAGTTCTACACCGAA
E G N W D L V G N N T P V F F I R D P I L F P S L I H T Q K
541 GAAGGGAACTGGGATTTGGTTGGCAACAACACACCCGTGTTCTTTATCCGAGACCCTATTCTTTTCCCTTCACTCATCCACACTCAGAAG
R N P A T H L K D P D M F W D F I T L R P E T T H Q V T I L
631 AGGAATCCAGCAACACACCTGAAGGACCCCGACATGTTCTGGGACTTCATAACCCTGAGACCTGAGACCACGCACCAGGTCACCATCCTT
F S D R G T P D G Y R H M N G Y G S H T F K L V N T E G K A
721 TTCTCGGACCGAGGTACTCCTGATGGCTACAGGCATATGAATGGTTATGGTTCTCACACTTTCAAGCTGGTGAATACTGAGGGAAAAGCC
V Y C K F H Y K T D Q G I R N L S A E K A D D L A G T D P D
811 GTTTACTGTAAATTCCACTACAAGACTGACCAAGGAATCAGAAATTTGAGCGCGGAGAAGGCCGACGACTTAGCTGGAACTGACCCTGAC
Y A I R D L Y N A I E G G D Y P S Y T M Y I Q V M T Y E E A
901 TATGCTATTCGTGATTTGTACAACGCTATTGAAGGCGGAGACTACCCATCATATACTATGTACATCCAGGTTATGACATATGAAGAGGCT
E K H R F N P F D L T K V W P H K D F P L I P V G R L T F D
991 GAGAAGCACCGGTTCAATCCTTTCGACTTGACCAAAGTGTGGCCACACAAGGATTTCCCTCTCATCCCTGTGGGACGCCTCACTTTTGAC
R N P R N Y F A E V E Q I A F S P A N M V P G I E A S P D K
1081 AGAAATCCTAGGAACTACTTTGCAGAGGTGGAACAGATTGCCTTTTCTCCAGCAAATATGGTCCCTGGCATCGAAGCATCACCCGACAAG
M L Q G R L F S Y N D T H R H R L G A N Y T Q I P V N C P Y
1171 ATGTTGCAAGGACGCCTTTTCTCCTACAATGACACCCATCGCCATAGGCTGGGCGCAAACTACACTCAGATACCTGTCAATTGTCCTTAT
R S R A R N Y Q R D G P M T V D Y N Q E S A P N Y F P N S F
1261 CGCTCTCGTGCTAGGAACTACCAGCGAGATGGGCCAATGACCGTCGATTACAACCAGGAAAGTGCTCCCAACTACTTCCCAAACAGCTTC
S G P M D C K R H S E S T F T C S T D V N R Y N S G D E D N
1351 TCTGGACCTATGGATTGTAAAAGGCATTCAGAGTCCACTTTCACTTGCTCGACCGACGTTAATCGGTACAACAGCGGCGATGAAGATAAC
F T Q A G N L F R H V M N E E E R Q R L V S N I A G H L V N
1441 TTTACTCAAGCTGGTAACTTGTTTAGACATGTCATGAACGAAGAGGAACGCCAGCGGCTAGTCAGCAACATTGCTGGTCACCTCGTGAAC
A Q K F L Q D R A I N N F S Q A D P E Y G A G I R R A L E K
1531 GCACAGAAGTTCCTGCAAGATCGTGCCATCAACAACTTCAGCCAAGCTGATCCTGAGTACGGTGCTGGAATTCGCAGGGCTCTTGAAAAA
L Q K A A A S H P V N L V A A P A S N A K L
1621 CTACAGAAGGCAGCAGCGTCTCACCCTGTGAACCTTGTAGCTGCTCCTGCTTCCAATGCCAAGCTATAAaaaaaaaaaa
```

Figure S1. Nucleotide and amino acid sequences of the full length cDNA of the *M. nipponense* catalase (*cat*) gene. The translated amino acid sequence is shown in standard one-letter code below the nucleotide sequence. The initiation codon and stop codon are indicated in the box.

```
1 gaaaagagtgttggaaattggctaaccggtaacaagtcagttgagagcgggcttttgtaaacaccggatcgcagaactctttcggttgtt
M S P
91 tttatcgtgcatttggctttaattaggtcttgtcagcacaattgtttggtgtattaccaatacccagttaatattctgcgaagATGTCGC
T L A Y W N I R G L A Q P I R L L L E Y T G T E F E D K M Y
181 CAACTCTTGCCTACTGGAACATTCGAGGTCTTGCCCAGCCTATTAGACTGCTGCTGGAATATACTGGAACTGAGTTTGAAGACAAGATGT
E C G P A P K F D K S C W F D I K Y S L G L D F P N L P Y Y
271 ATGAATGTGGACCAGCCCCAAAGTTTGACAAGTCTTGCTGGTTTGATATTAAGTACTCCTTAGGCCTCGACTTTCCCAACCTCCCTTACT
I D G D V K V T Q S N A I M R Y I A R K H D L C G K T E S E
361 ACATTGATGGAGATGTGAAAGTAACCCAGAGCAATGCCATTATGCGGTACATTGCAAGAAAGCATGATCTATGTGGCAAGACTGAATCTG
K V R V D I M E N Q S M D F R N G F V R L C Y V D F D T Q K
451 AGAAAGTCCGTGTTGATATTATGGAAAACCAATCAATGGACTTCAGGAATGGTTTTGTTCGACTGTGCTATGTAGATTTTGATACCCAGA
Q T Y L E A L P T T L K L F S T F L G E Q P W Y A G D N I T
541 AGCAGACCTACTTAGAGGCTTTACCCACTACATTGAAGCTGTTCTCAACATTCCTTGGTGAACAACCCTGGTATGCTGGTGATAATATCA
F V D F I M Y E L I D Q H L Q L D P N L L K D F K N L E D F
631 CTTTTGTGGATTTCATTATGTATGAACTTATTGATCAGCATCTGCAATTAGACCCAAACCTACTTAAGGATTTCAAGAACCTTGAAGACT
Q K R F E E L E P I K K Y M A S S R F M K S P L N N K M A K
721 TCCAGAAGCGTTTTGAAGAATTAGAACCCATCAAGAAGTACATGGCCTCCTCACGATTCATGAAGTCTCCACTTAATAACAAAATGGCAA
F G N
811 AGTTTGGAAACTAActttgttgtgaagaccataaatgttttaactttttttgcttacaatacaaatgtgcagcattataagtgataaaag
901 ttcctaatttagtgagtacatatatgctaaagaaaatagtaatttccagtttgacgtctgttatattatactaggtaaaccaaatatatg
991 ataaatattgtttctcggaatagtgtaattttgtttttgagaaattgtgcaaaatttatttgaaggtcttatttgaagacactgtttgat
1081 ataaacagctttgaaataaaaggtttaaagtaaaaaaaaaaa
```

Figure S2. Nucleotide and amino acid sequences of the full length cDNA for glutathione *S*-transferase (*gst*) in *M. nipponense*. The translated amino acid sequence is shown in standard one-letter code below the nucleotide sequence. The initiation codon and stop codon are indicated in the box. The polyadenylation signal is shaded light gray.

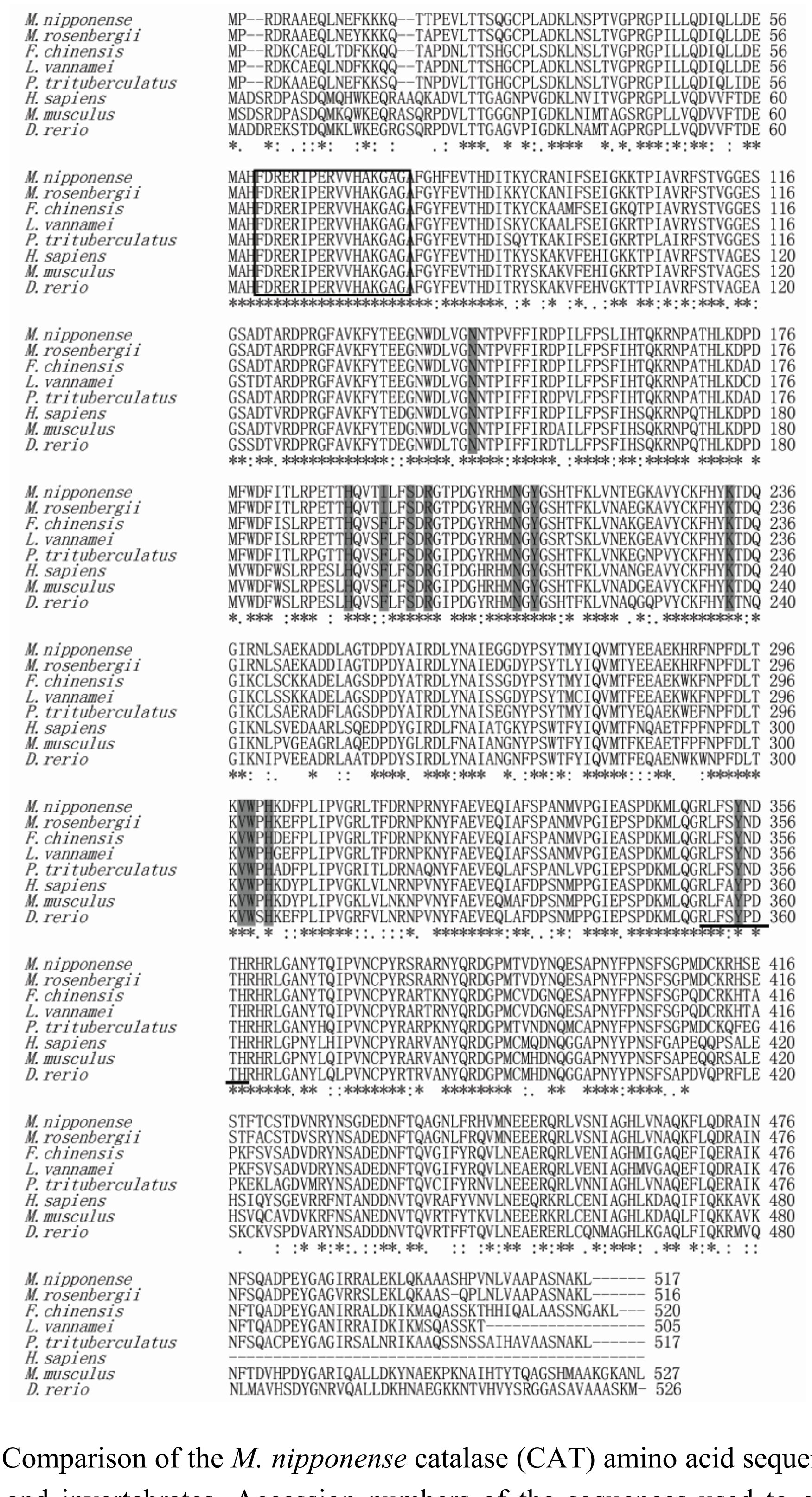

Figure S3. Comparison of the *M. nipponense* catalase (CAT) amino acid sequences among vertebrates and invertebrates. Accession numbers of the sequences used to compare the amino acid residues are as follows: *Danio rerio*, NM130912; *Homo sapiens*, AY028632; *Hyriopsis cumingii*, HM188565; *Eriocheir sinensis*, GU361618; *Macrobrachium rosenbergii*, HQ668089; and *Litopenaeus vannamei*, JX162772. The CAT proximal heme-ligand signature is framed. Shaded region indicates amino acid residues combined with coenzyme (NADPH). Underline indicates CAT proximal active signature site. Asterisks indicate positions where all sequences share the same amino acid residues.

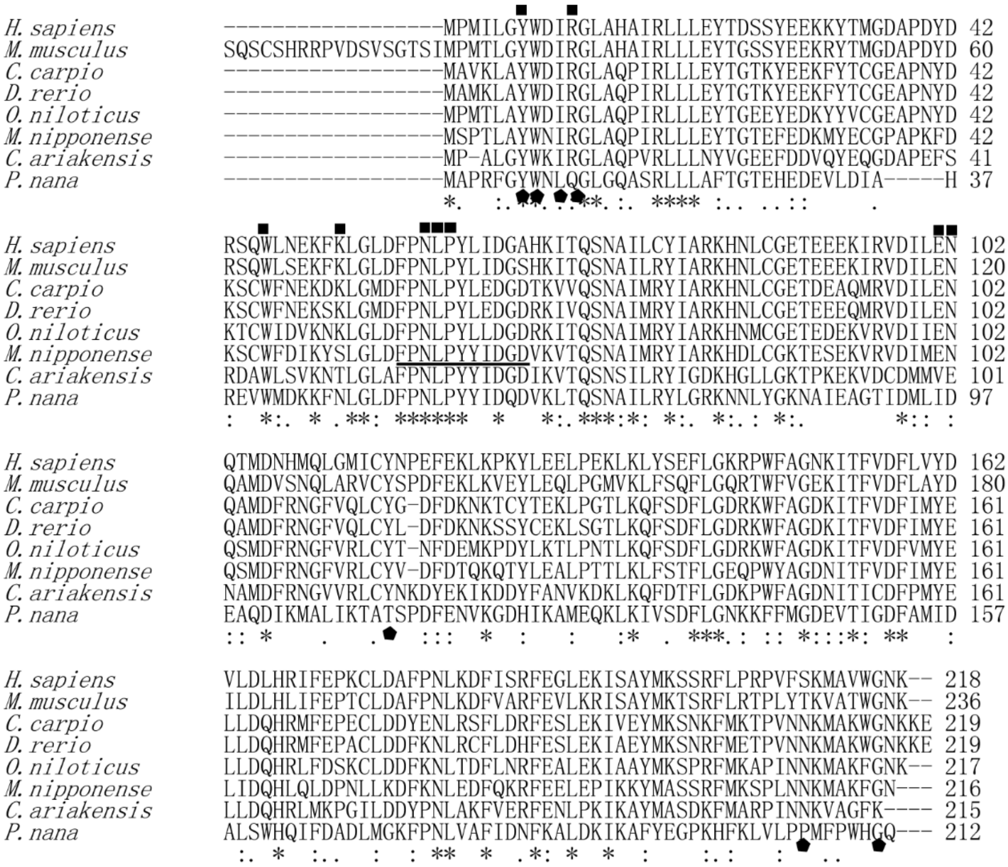

Figure S4. Comparison of *M. nipponense* glutathione *S*-transferase (GST) amino acid sequences among vertebrates and invertebrates. Accession numbers of the sequences used to compare amino acid residues are as follows: *Homo sapriens*, M63509; *Mus musculus*, X65021; *Cyprinus carpio*, DQ411212; *Oreochromis niloticus*, EU107284; *Crassostrea ariakensis*, EU908270 and *Paracyclopina nana*, HQ115574. "■" indicate G-sites and "⬟" indicate H-sites. Symbols below the amino acid sequences indicate invariant (*), strongly conserved (:) or conserved residues (.).
